# Mosquito-Associated Viruses in China

**DOI:** 10.1007/s12250-018-0002-9

**Published:** 2018-03-12

**Authors:** Han Xia, Yujuan Wang, Evans Atoni, Bo Zhang, Zhiming Yuan

**Affiliations:** 1 Key Laboratory of Special Pathogens and Biosafety, Wuhan Institute of Virology, Chinese Academy of Sciences, Wuhan, 430071 China; 2 University of Chinese Academy of Sciences, Beijing, 100049 China

**Keywords:** Mosquito associated viruses, China, Vector, Public health

## Abstract

Mosquitoes are classified into approximately 3500 species and further grouped into 41 genera. Epidemiologically, they are considered to be among the most important disease vectors in the world and they can harbor a wide variety of viruses. Several mosquito viruses are considered to be of significant medical importance and can cause serious public health issues throughout the world. Such viruses are Japanese encephalitis virus (JEV), dengue virus (DENV), chikungunya virus (CHIKV), and Zika virus (ZIKV). Others are the newly recognized mosquito viruses such as Banna virus (BAV) and Yunnan orbivirus (YNOV) with unclear medical significance. The remaining mosquito viruses are those that naturally infect mosquitoes but do not appear to infect humans or other vertebrates. With the continuous development and improvement of mosquito and mosquito-associated virus surveillance systems in China, many novel mosquito-associated viruses have been discovered in recent years. This review aims to systematically outline the history, characteristics, distribution, and/or current epidemic status of mosquito-associated viruses in China.

## Introduction

Mosquitoes are small insects that are taxonomically classified into the family *Culicidae*. There are approximately 3500 species of mosquito in the world, grouped into 41 genera. Mosquitoes carry a wide variety of viruses, and these viruses can be classified into two categories: mosquito-borne viruses and mosquito-specific viruses. Mosquito-borne viruses are arboviruses, which can replicate within the mosquito but can also be transmitted biologically to vertebrates and infect vertebrate cells. Most of the mosquito-borne viruses are pathogenic viruses responsible for disease outbreaks in human and animal populations, such as dengue virus (DENV), chikungunya virus (CHIKV), yellow fever virus (YFV), Zika virus (ZIKV), and Akabane virus (AKV). The majority of mosquito-borne viruses are distributed within the families *Peribunyaviridae*, *Flaviviridae*, *Togaviridae*, and *Reoviridae*. Mosquito-specific viruses, as insect-specific viruses (ISVs), can infect mosquitoes naturally and replicate in mosquito cells *in vitro*, but do not replicate in vertebrate cells or infect humans or other vertebrates (Bolling *et al.* 2015). These viruses are distantly related to known pathogenic viruses and are found within the families *Parvoviridae*, *Flaviviridae*, *Togaviridae*, *Rhabdoviridae*, *Peribunyaviridae*, *Reoviridae*, *Mesoniviridae*, *Tymoviridae*, and *Birnaviridae*.

With the development and improvement of mosquito and mosquito-associated virus surveillance systems in China, many novel mosquito-associated viruses have been discovered and identified in recent years. This review briefly summarizes the present knowledge about the mosquito-associated viruses in mainland China (Table 1).

**Table 1:** Mosquito-associated viruses and their characters.

Type	Family	Virus	Mosquito source for virus	Prevalence status of related viral disease to human or animal	Geographic Distribution of the virus	References
Mosquito-borne virus pathogenic to human or animal	*Flavivirade*	Dengue virus (DENV)	*Aedes aegypti, Aedes albopictus*	Imported case (human)	Guangdong, Hainan, Guangxi, Fujian, Zhejiang, Jiangsu, Yunnan, Henan, Hubei, Beijing	Wang W *et al.* (2015), Wu *et al.* (2010), Xiong and Chen (2014), Zhang *et al.* (2014), Zhao *et al.* (2016)
		Japanese encephalitis virus (JEV)	*Culex tritaeniorhynchus*, *Aedes vexans, Culex modestus, Culex pipiens pallens, Culex theileri, Anopheles sinensis*, *Armigeres subalbatus*	Endemic (human)	Except Xinjiang, and Qinghai	Gao *et al.* (2015), Li *et al.* (2011), Wang HY *et al.* (2009), Wang *et al.* (2007)
		Tembusu virus (TMUV)	*Culex pipiens, Culex tritaeniorhynchus*	Endmic (duck)	Beijing, Hebei, Shandong, Jiangsu, Anhui, Zhejiang, Jiangxi, Fujian, Guangdong, Guangxi, Chongqing, Shanghai, Yunnan	Cao ZZ *et al.* (2011), Lei *et al.* (2017), Tang *et al.* (2015), Yan *et al.* (2011), Zhang *et al.* (2017)
		West Nile virus (WNV)	*Culex pipiens*	Sporadic case (human)	Xinjiang	Jiang *et al.* (2010, 2014), Li *et al.* (2013), Lu Z *et al.* (2014)
		Yellow fever virus (YFV)	*NA*	Imported case (human)	Beijing, Fujian, Shanghai, Jiangxi	Chen and Lu (2016), Chen *et al.* (2016), Ling *et al.* (2016), Wasserman *et al.* (2016)
		Zika virus (ZIKV)	*Culex quinquefasciatus, Armigeres subbalbeatus*	Imported case (human)	Beijing, Guangdong, Zhejiang, Henan, Guizhou	Deng YQ *et al.* (2016), Deng C *et al.* (2016), Su *et al.* (2016), Zhang *et al.* (2016), Fu *et al.* (2017)
	*Peribunyaviridae*	Batai virus (BATV)	*Anopheles philippinensis*	Infection (human)	Yunnan, Inner Mongolia	Huang *et al.* (2001), Liu *et al.* (2014), Wang FT *et al.* (2009)
		Tahyna virus (TAHV)	*Aedes vexans, Aedes detritus, Culex* spp.	Sporadic case (human)	Xinjiang, Qinghai, and Inner Mongolia	Cao YX *et al.* (2011), Li W *et al.* (2014), Lu *et al.* (2009, 2011a), Lv *et al.* (2011)
	*Phenuiviridae*	Rift Valley fever virus (RVFV)	NA	Imported case (human)	Beijing	Liu *et al.* (2017), Shi *et al.* (2017)
	*Reoviridae*	Banna virus (BAV)	*Culex tritaeniorhynchus, Culex pipiens pallens, Culex annulus, Culex pseudovishnui, Culexmodestus, Anopheles sinensis, Aedes vagus, Aedes albopictus, Aedes vexans, Aedes dorsalis*	Sporadic case (human)	Gansu, Guizhou, Liaoning, Beijing, Yunnan, Shanxi, Inner Mongolia	Liu *et al.* (2010), Wang *et al.* (2011), Xu *et al.* (1990)
		Liaoning virus (LNV)	*Aedes dorsalis, Aedes caspius, Aedes flavidorsalis, Culex modestus, Culex pipiens*	NA	Liaoning, Qinghai, Xinjiang, Beijing, Shanxi, Gansu	Attoui *et al.* (2006), Li *et al.* (2010), Liu *et al.* (2011), Lu *et al.* (2011b), Lv *et al.* (2012)
		Yunnan orbivirus (YNOV)	*Culex tritaeniorhynchus, Culicoides* spp.,* Anopheles maculatus*	Infection (human)	Yunnan	Attoui *et al.* (2005b, 2009), Wang *et al.* (2011), Zhang and Liang (2012)
		Tibet orbivirus (TIBOV)	*Culicoides* spp.,* Culex fatigan, Culex tritaeniorhynchus*	NA	Tibet, Yunnan, Guangdong	Li M *et al.* (2014), Lei *et al.* (2015), Xing *et al.* (2017)
	*Togaviridae*	Chikungunya virus (CHIKV)	*Aedes albopictus*	Imported case (human)	Yunnan, Guangdong, Zhejiang	Huang *et al.* (1998), Zhang *et al.* (2012), Sun *et al.* (2013), Wu *et al.* (2012), Zheng *et al.* (2010)
		Getah virus (GETV)	*Aedes vexans, Armigeres obturbans, Armigeres subalbatus, Culex* spp.	Infection (human, horse, pig)	Hainan, Hebei, Yunnan, Shanghai, Shanxi, Gansu	Li *et al.* (2017), Liu *et al.* (2011), Wang *et al.* (2011), Zhai *et al.* (2008a)
		Ross River virus (RRV)	*NA*	Infection (human, rats)	Hainan, Guizhou	Ye *et al.* (2012), Zhao *et al.* (1997)
		Sindbis virus (SINV)	*Anopheles* spp.	Infection (human)	Yunnan, Xinjiang	Liang *et al.* (2000), Wang *et al.* (2008), Zhang and Liang (2012), Zhou *et al.* (1999)
Mosquito-specific virus	*Flavivirade*	Chaoyang virus (CHAOV)	*Aedes vexans*	NA	Liaoning	Liu *et al.* (2011), Wang ZS *et al.* (2009)
		Culex favivirus (CxFV)	*Culex Pipiens, Culex Tritaeniorhynchus, Anopheles Sinensis*	NA	Shandong, Henan, and Shaanxi, Liaoning, Yunnan	Wang *et al.* (2012), Liang *et al.* (2015), Zuo *et al.* (2014)
	*Mesoniviridae*	Nam Dinh virus (NDiV)	*Culex pipiens quinquefasciatus*	NA	Guangdong	Zhou *et al.* (2017)
		Yichang virus (YCV)	*Culex tritaeniorhynchus*	NA	Hubei	Wang *et al.* (2017)
	*Parvoviridae*	Culex pipienspallensdensovirus (CppDNV)	*Culex pipiens pallens, Aedes caspius, Anopheles vagus*	NA	Liaoning, Inner Mongolia, Qinghai, Shanxi, Yunnan, Xingjiang, Guizhou	Cao YX *et al.* (2011), Feng *et al.* (2014), Li *et al.* (2010), Zhai *et al.* (2008b), Zheng *et al.* (2015)
	*Reoviridae*	Kadipiro virus (KDV)	*Culex tritaeniorhynchus, Anopheles sinensis, Armigeres obturbans*	NA	Yunnan	Liu *et al.* (2011), Sun *et al.* (2009a, b)
		Mangshi virus (MSV)	*Culex* *tritaeniorhynchus*	NA	Yunnan	Wang J *et al.* (2015)

## Mosquito-Borne Viruses Pathogenic to Humans or Animals

### *Flaviviridae*

#### *Dengue virus* (DENV)

DENV is a member of the genus *Flavivirus*, family *Flaviviridae*. It is related to other medically important arboviruses such as YFV and Japanese encephalitis virus (JEV).

DENV was first isolated from human blood samples in Japan, in the year 1943 (Messina *et al.* 2014). Since then, four phylogenetically and antigenically distinct DENVs (DENV1–4) have been identified. In China, all four serotypes of DENV have caused dengue outbreaks. DENV-1 has been the primary epidemic serotype since the 1990s (Lai *et al.* 2015; Xiong and Chen 2014). DENV-4 was the first documented serotype in Guangdong in 1978, with a second DENV-4 outbreak occurring in 1990. In the year 2010, after an absence of almost 20 years, DENV-4 was again detected during an outbreak in Guangzhou and initially traced back to an imported case: a Guangzhou resident who had traveled back home from Thailand (Jing *et al.* 2012). DENV-3 was recorded in Hainan in 1980. Later, DENV-3 was also isolated in outbreaks in Guangdong in 2009 and 2010, Zhejiang in 2009 (Yan *et al.* 2010), and Yunnan and Henan in 2013 (Huang *et al.* 2014; Zhang *et al.* 2014). DENV-2 was confirmed in Hainan in 1985–1987, and a few cases were reported in 2013 and 2014 (Lai *et al.* 2015; Xiong and Chen 2014).

DENV is carried and spread by mosquitoes of the genus *Aedes* and mainly through *Aedes aegypti*, which is active in the living spaces of humans. *Aedes aegypti* and *Aedes albopictus* are the primary vectors of DENV in China. Imported DENVs may infect the vector population during permissive climatic conditions (Wu *et al.* 2010).

Symptoms for DENV infection typically include a high fever, headache, vomiting, muscle and joint pains, and skin rash. In a small proportion of cases, the disease develops into a serious dengue hemorrhagic fever/dengue shock syndrome (DHF/DSS) resulting in high mortality (Heilman *et al.* 2014). Currently, there is no US Food and Drug Administration (FDA)-approved vaccine or antiviral treatment specifically for dengue. In China, during 1978–1991, the epidemic regions were mainly in Guangdong and Hainan. After 1990, the endemic range of dengue fever in China expanded geographically, with the new cases being reported in Guangxi, Fujian, Zhejiang, Jiangsu, Yunnan, Henan, Hubei, and Beijing. In 2013, the first dengue fever outbreak in central China occurred in Henan province (Wang W *et al.* 2015; Zhang *et al.* 2014). These outbreaks of dengue fever in China are suspected to result from virus imported by infected travelers coming from dengue-endemic regions or local epidemics initiated by imported cases. Recently, dengue fever has shown an increasing drift especially in the four main hotspot areas, namely Guangdong, Fujian, Zhejiang, and more recently Yunnan (Gu *et al.* 2012; Sun *et al.* 2011; Yan *et al.* 2010; Yang *et al.* 2015; Zhang *et al.* 2014; Zhao *et al.* 2016). According to the China National Notifiable Disease Surveillance System, from the year 2002–2016, the incidence ranged from 0.01 to 3.46 per 100,000 people, with a total of seven deaths reported. In the year 2014, there were more than 44,000 reported cases of dengue fever, which stands out as the highest ever number of dengue infections per year in the historical records.

#### *Japanese encephalitis virus* (JEV)

JEV is a member of the Japanese encephalitis (JE) serogroup of the genus *Flavivirus*, family *Flaviviridae*. The prototype Nakayama strain was first isolated in 1953 from the brain of a fatal case and has subsequently been found across most parts of Asia (Solomon *et al.* 2000). JEV was first isolated from human brain (strains A2 and P3) in China in the 1940s, and since then JE epidemics have occurred in China for over 60 years (Wang *et al.* 2007, Wang HY *et al.* 2009). Phylogenetic analyses based on the E region divides JEV into five genotypes (Gao *et al.* 2015; Solomon and Ni 2003; Wang *et al.* 2007). Molecular biological research has indicated that the JEV strains isolated in China are mainly divided into two genotypes: genotypes 1 and 3. Genotype 1 JEV strains have been isolated since 1979, while genotype 3 JEV strains have been isolated since the 1940s, and genotype 1 has gradually been replacing genotype 3 as the dominant genotype in the JE outbreaks during the last decade (Gao *et al.* 2015; Wang *et al.* 2007, Wang HY *et al.* 2009). One strain of genotype 5 JEV (strain XZ0934) was isolated from *Culex tritaeniorhynchus* collected in Tibet in 2009 (Li *et al.* 2011).

JEV has a zoonotic transmission cycle between mosquitoes and vertebrate hosts. *Culex tritaeniorhynchus* is the most important vector for JEV in China. However, other mosquito species have also been implicated as vectors of JEV, such as *Aedes vexans*, *Culex modestus*, *Culex pipiens pallens*, *Culex theileri*, *Anopheles sinensis*, and *Armigeres subalbatus* (Wang *et al.* 2007). In nature, the virus cycle is maintained between mosquitoes and vertebrate hosts such as bats, water birds, and pigs.

The human disease caused by JEV results in inflammation of the brain, with other symptoms including high fever, coma, tremors, and convulsions. The mortality rate is 5%–40% (Misra and Kalita 2010). Occurrence and epidemic outbreaks of JEV have been reported in all provinces of mainland China except Xinjiang, Tibet, and Qinghai. Based on seasonal distribution, JE cases in the south tend to increase during the month of July and decrease significantly in August, whereas in the north, they begin to increase in the month of August and dramatically decrease in September (Wang HY *et al.* 2009). The highly endemic areas (with average incidence > 1/100,000) include Sichuan, Guizhou, Chongqing, Shaanxi, and Yunnan, which are located in southwest and central China and adjacent to each other. The moderately endemic regions (with average incidence of between 0.5/100,000 and 1/100,000) include Shanxi, Henan, Anhui, Hubei, Hunan, Jiangxi, and Guangxi (Wang HY *et al.* 2009). A large epidemic of JE occurred in the 1960s and early 1970s, and a total of 170,000 cases were reported with incidence rates of 20/100,000 people and a fatality rate of 25% (Gao *et al.* 2010). The development of a JE live attenuated vaccine (SA14-14-2) in 1989 and its expansive use thereafter has seen the number of JE cases, especially the fatal cases, reduced significantly. For instance, the incidence rates from 2002 to 2016 remained at 1/100,000 people.

#### *Tembusu virus* (TMUV)

TMUV is a member of the Ntaya virus serogroup in the genus *Flavivirus*, family *Flaviviridae*. TMUV was first isolated in 1955 from *Culex tritaeniorhynchus* mosquitoes in Kuala Lumpur, Malaysia. Since then, it has been successively isolated from mosquitoes (*Culex spp.*) and a variety of avian species including ducks, geese, chickens, pigeons, and sparrows in Southeast Asia or China (Zhang *et al.* 2017). In China, it was first isolated from ducks in an outbreak of duck egg-drop disease in 2010 (Cao ZZ *et al.* 2011; Yan *et al.* 2011). Phylogenetic analysis indicated that TMUV did not exhibit a species barrier in avian species and consisted of two lineages: the Southeast Asian and the Chinese lineages (Lei *et al.* 2017).

In nature, the cycle for TMUV-mosquito-avian transmission remains unclear. However, vector competence tests showed that field-caught mosquitoes can be efficient vectors for spreading the virus (O’Guinn *et al.* 2013). The detection and isolation of TMUV from mosquitoes (*Culex pipiens* and *Culex tritaeniorhynchus*) in China was reported in Shandong and Yunnan provinces, and the results showed a high percentage of positives for TMUV by both RT-PCR and virus isolation assays (Lei *et al.* 2017; Tang *et al.* 2015).

TMUV-associated diseases in animals have been noted in China and Malaysia, such as encephalitis and retarded growth in broiler chicks (Kono *et al.* 2000) and duck egg-drop disease (Cao ZZ *et al.* 2011; Yan *et al.* 2011). In China, since 2010, duck egg-drop disease has been reported in most duck-producing regions, including Beijing, Hebei, Shandong, Jiangsu, Anhui, Zhejiang, Jiangxi, Fujian, Guangdong, Guangxi, Chongqing, and Shanghai (Zhang *et al.* 2017). In addition, a serological survey in Shangdong suggested there were human infections by TMUV, but there are no current reports of human disease related to TMUV (Tang *et al.* 2013).

#### *West Nile virus* (WNV)

WNV is a member of the genus *Flavivirus*, family *Flaviviridae*. WNV was first isolated in a woman in the West Nile district of Uganda in 1937. Since then, it has been detected in Africa, Asia, Europe, Australia, and North America (Chancey *et al.* 2015). In China, WNV was first isolated from *Culex pipiens* mosquitoes in Xinjiang in 2011. The Xinjiang WNV isolates showed a high degree of genetic similarity to lineage 1 containing other highly pathogenic WNV strains (Lu Z *et al.* 2014).

In nature, WNV cycles between mosquitoes and birds, and almost 75 mosquito species have been identified as WNV vectors (mainly the *Culex* species), with different species recognized as primary vectors in different areas (Rossi *et al.* 2010). Humans, horses, and other mammals can be infected through mosquito bites. In addition, four species of *Culex* mosquitoes found in China were determined to be competent laboratory vectors of WNV (Jiang *et al.* 2010, 2014).

Most people infected with WNV develop a fever with other symptoms such as headache, body aches, joint pains, and vomiting, and less than 1% of people will develop a serious neurologic disease such as encephalitis or meningitis (Chancey *et al.* 2015; Rossi *et al.* 2010). Currently, no vaccine or specific antiviral treatments for WNV infection are available. In China, seropositivity for WNV was first reported in birds from Yunnan province in 1988. Serological evidence was also reported in cats, dogs, and captive resident birds in Shanghai in 2010 (Lan *et al.* 2011). The first confirmed human cases of WNV in China were reported in 2013 during an outbreak of fever and meningitis/encephalitis in Xinjiang in 2004 (Li *et al.* 2013). Since then, no new cases of WNV in China have been reported. Subsequently, evidence of WNV human infections was confirmed by IgM ELISA and seroconversion by 90% plaque reduction neutralization test of paired serum samples obtained from persons with febrile illness and viral encephalitis in Xinjiang in 2004 (Lu Z *et al.* 2014). Even though reported WNV cases have been limited in Xingjiang until now, it is possible that migrating birds and the domestic animal trade could introduce WNV from endemic regions to China or induce local transmission in China.

#### *Yellow fever virus* (YFV)

YFV is the prototype virus of the genus *Flavivirus*, family *Flaviviridae*, which causes yellow fever with symptoms ranging from mild, non-specific febrile illness to a severe acute disease with hemorrhage, vomiting, renal failure, and severe hepatic dysfunction in humans (Monath and Vasconcelos 2015). YFV was first isolated in 1927 from a Ghanaian patient named Asibi in West Africa. YFV is known to be widely distributed in tropical regions of Africa and South America (Jentes *et al.* 2011; Monath and Vasconcelos 2015). YFV can be transmitted to humans and non-human primates through mosquito bites. *Aedes aegypti* is the only vector in the “urban cycle” known to be responsible for the major outbreaks of yellow fever in Africa. In the sylvatic “jungle cycle”, monkeys act as the host and *Aedes africanus* and other *Aedes spp.* are the vectors (Chen and Lu 2016).

No YFV isolate or yellow fever case was reported in China before 2016 (Wasserman *et al.* 2016). In China, YFV was first (strain CNYF01/2016) isolated from the first imported case in Beijing in 2016. Phylogenetic analysis showed the strain belongs to the Angola71 genotype, which has only 14 amino-acid substitutions throughout the genome and no amino-acid changes observed in the membrane or envelope proteins compared with the YFV strain collected in 1971 (Chen *et al.* 2016). By the end of 2016, there were a total of 11 confirmed imported cases of yellow fever, of which five were identified in Beijing, another five cases were reported in Fujian, and one case was identified in Shanghai (Ling *et al.* 2016; Wasserman *et al.* 2016). All the patients had traveled back to China from Angola. Although no local case of yellow fever has been reported, with the growing migration in southern China, where the *Aedes aegypti* density is relatively high, there is an increased risk of autochthonous transmission in this region (Chen and Lu 2016). The main strategies for control of yellow fever transmission in China entail continuous screening, control of vectors, vaccination of travelers that are going to yellow fever endemic regions, and strengthening of public education.

#### *Zika virus* (ZIKV)

ZIKV belongs to the genus *Flavivirus*, family *Flaviviridae*. It was first isolated in 1947 from a rhesus monkey in Zika Forest, Uganda. Human infections with Zika virus (ZIKV) usually present mild symptoms such as fever, skin rashes, muscle and joint pain, and headache. But recently, the infection was reportedly associated with microcephaly in infants and neurological disorders such as Guillain–Barré syndrome (GBS) (Rasmussen *et al.* 2016; Zanluca *et al.* 2016). ZIKV had limited circulation in Africa, Southeast Asia, and the Pacific prior to the year 2015. Since then, it has rapidly spread to more than 30 countries in Africa, the Americas, Asia, and the Pacific (Bogoch *et al.* 2016). ZIKV is classified into two major lineages, the African and Asian lineages. *Aedes aegypti* and *Aedes albopictus* are the two major mosquito vectors responsible for ZIKV transmission.

The first Chinese ZIKV strain was isolated from an imported case in Shenzhen in 2016 (Deng C *et al.* 2016; Deng YQ *et al.* 2016). By the end of 2016, a total of 22 ZIKV disease cases were reported in China, 14 cases in Guangdong, two cases in Beijing, four cases in Zhejiang, one case in Henan, and one case in Jiangxi. All these patients had recent travel history to South America or Oceania (Zhang *et al.* 2016). Seven available Chinese imported ZIKV sequences cluster within the Asian lineage, separated into three groups, and demonstrate a high genetic diversity. Since the *Aedes* mosquitoes are broadly distributed in southern China, where warm weather and precipitation are ideal for promoting the population growth of *Aedes*, the risk of imported ZIKV spreading within these regions is increasing (Su *et al.* 2016). In addition, two ZIKV strains were isolated from *Culex quinquefasciatus* and *Armigeres subbalbeatus* respectively in a survey in Guizhou province, in August, 2016 (Fu *et al.* 2017). Phylogenetic analyses indicated that the strains (GZDJ1685) from *Culex quinquefasciatus* was clustered into the Asian branch along with isolates from Brazil (2015), Puerto Rico (2015), and Yap Island (2007) (Song *et al.* 2017). This is the first report of ZIKV isolation in nature in China and presents new challenges for the prevention and control of ZIKV in China (Fu *et al.* 2017; Song *et al.* 2017).

### *Peribunyaviridae*

#### *Batai virus* (BATV)

BATV is a member of the Bunyamwera group in the genus *Orthobunyavirus*, family *Peribunyaviridae*. BATV was originally isolated (MM-2222) from *Culex gelidus* collected in Malaysia in 1955 (Karabatsos 1978). BATV is widely distributed throughout large parts of Africa, Asia, and Europe. In China, BATV (strain YN92-4) was first isolated from an *Anopheles philippinensis* mosquito found in Yunnan in 1988 (Wang FT *et al.* 2009). In 2012, BATV (strain NM/12) was isolated from cattle in Inner Mongolia (Liu *et al.* 2014). Phylogenetic analyses based on the S, M, and L segments revealed that the YN92-4 strain belongs to the same group as the MM-2222 strain isolated in Malaysia (Wang FT *et al.* 2009), and the NM/12 strain is closely related to strains found in different regions of Asia and distantly related to European BATV strains (Liu *et al.* 2014).

BATV is transmitted by the mosquitoes *Anopheles maculipennis* s.l., *Anopheles claviger*, and *Ochlerotatus spp.* The vertebrate hosts of BATV include domestic pigs, horses, ruminants, and wild birds (Bialonski *et al.* 2011; Huhtamo *et al.* 2013; Liu *et al.* 2014).

Human infection by BATV shows symptoms of self-limiting influenza-like febrile illness. In addition, BATV has been implicated as a segment donor in the generation of the reassortant Ngari virus which caused outbreaks of hemorrhagic illness in East Africa (Briese *et al.* 2006; Liu *et al.* 2011). A sero-survey in Xishuangbanna in Yunnan showed that the positive rate of BATV specific antibody was 4.7% (5/120) in febrile patients, giving evidence of BATV infection in this locality (Huang *et al.* 2001).

#### *Tahyna virus* (TAHV)

TAHV is a member of the California antigenic group in the genus *Orthobunyavirus*, family *Peribunyaviridae* (Hubálek 2008; Lu *et al.* 2009). TAHV was originally isolated (prototype strain T-92) from a pool of *Aedes caspius* mosquitoes in eastern Slovakia in 1958 (Hubálek 2008). TAHV occurs in Asia, Africa, and Europe. In China, it was first isolated (strain XJ0625) from *Culex spp.* in Kashi, Xinjiang, in 2006 (Lu *et al.* 2009). Subsequently, TAHV has been isolated from Xinjiang, Qinghai, and Inner Mongolia (Cao YX *et al.* 2011; Li W *et al.* 2014; Lu *et al.* 2011a). Phylogenetic analysis generated from N segment and polymerase gene sequences indicated that all TAHV isolates from China group together. However, the analysis for the M segment showed the isolate XJ0708 aligns independently within the context of a relatively large diversity of isolates from Europe (Lu *et al.* 2011a).

TAHV is maintained in several species of mosquito vectors and vertebrates such as hares, rabbits, hedgehogs, and rodents. In China, TAHV has been isolated from *Aedex vexans*, *Aedes detritus*, and *Culex spp.* (Li W *et al.* 2014; Lu *et al.* 2009, 2011a).

The human disease caused by TAHV is an influenza-like illness with a sudden onset of fever, or pneumonia, acute arthritis, and rarely meningoencephalitis. Currently, no mortality has been reported as a result of TAHV infection. TAHV antibodies (IgG) have been detected in serum from fever and headache patients in Jiashi County in Xinjing with a 13.0% positive rate, and in local livestock in Geermu city in Qinghai (Li W *et al.* 2014; Lu *et al.* 2009; Lv *et al.* 2011). In 2009, confirmed cases of TAHV were reported for the first time in Qinghai (Li W *et al.* 2014).

### *Phenuiviridae*

#### *Rift Valley fever virus* (RVFV)

RVFV is the member of genus *Phlebovirus*, family *Phenuiviridae.* The RVFV genome (∼ 12 kb) consists of three negative-stranded RNA, designated large (L), medium (M) and small (S) segment, and the viruses can be classified into seven major genetic lineages (Pepin *et al.* 2010; Fu *et al.* 2016). RVFV could cause high levels of mortality and morbidity in domesticated animals and mild to serious disease in humans. RVFV was first isolated from sheep in Kenya in 1930s, subsequently it is present or endemic in Africa, Saudi Arabia and Yemen.

The main vectors of RVFV transmission are the mosquitoes in the genera of *Aedes spp.* and *Culex spp.*, and the outbreaks of Rift Valley fever are strong correlated with heavy rainfall or flooding (Pepin *et al.* 2010; Fu *et al.* 2016).

Recently, the first imported case of Rift Valley fever was confirmed China on 23 July, 2016. The patient was a 45-year-old male who returned from Angola and arrived in Beijing Capital International Airport. The RVFV was isolated from the patient’s blood, and the whole-genome sequencing and phyleogenetic analysis indicated that the imported strain was a reassotant comprising the L and M segments from lineage E and the S segment from lineage A (Liu *et al.* 2017; Shi *et al.* 2017).

### *Reoviridae*

#### *Banna virus* (BAV)

BAV is the prototype species of the genus *Seadornavirus* that includes Kadipiro virus (KDV) and Liaoning virus (LNV) within the family *Reoviridae* (Attoui *et al.* 2000, 2005b; Liu *et al.* 2010). The BAV genome is around 21 kb, which consists of 12 segments of double-stranded RNA (dsRNA) (Liu *et al.* 2010). BAV (strain BAV-Ch) was originally isolated from a patient with encephalitis and fever in Yunnan, China, in 1987 (Xu *et al.* 1990). BAV is widely distributed in China (Gansu, Liaoning, Beijing, Yunnan, Shanxi, Inner Mongolia) and Southeast Asia (Indonesia, Vietnam) (Liu *et al.* 2010; Nabeshima *et al.* 2008; Wang *et al.* 2011).

In nature, BAV can be detected and isolated from pigs, cattle, and ticks (Liu *et al.* 2010; Wang *et al.* 2011). To date, BAV isolates have been obtained from 10 mosquito species in three genera (*Culex tritaeniorhynchus*, *Culex pipiens pallens*, *Culex annulus*, *Culex pseudovishnui*, *Culex modestus*, *Anopheles sinensis*, *Aedes vagus*, *Aedes albopictus*, *Aedes vexans*, and *Aedes dorsalis*).

Phylogenetic analysis of BAV, according to segment 12, divides the virus strains into two different groups according to their geographic origin: isolates from China and Vietnam are included in group A, and the strains from Indonesia are in group B (Liu *et al.* 2010, 2016).

BAV is suspected to be an encephalitis-causing pathogen in humans. The clinical symptoms of BAV infection are inflammation of the brain and fever, which is similar to JE. A large-scale sero-survey of patients supposed to have had JE or viral encephalitis was conducted in several provinces in China. In this survey, the indirect enzyme linked immunosorbent assay (ELISA) results indicated that the positive rate of anti-BAV IgM antibodies was 11.4%. However, no fourfold or greater BAV specific antibody response was detected by neutralization test in serum from the acute or convalescent phase of illness (Tao and Chen 2005). Further investigation of the association between BAV and human diseases should be conducted (Liu *et al.* 2011).

#### *Liaoning virus* (LNV)

LNV is a member of the genus *Seadornavirus* within the family *Reoviridae*, which is composed of 12 segments of dsRNA. LNV was first obtained from *Aedes dorsalis* mosquitoes in Liaoning province in China in 1997 (strains LNV-NE9712 and LNV-NE9731) (Attoui *et al.* 2006). Subsequently, LNV isolates from mosquitoes have been obtained from many regions in China, such as Xinjiang, Qinghai, Beijing, Shanxi, and Gansu, and from several mosquito species including *Aedes dorsalis*, *Aedex caspius*, *Aedes flavidorsalis*, *Culex modestus*, *Culex pipiens*, *and Culex spp.*. (Li *et al.* 2010; Liu *et al.* 2011; Lu *et al.* 2011b; Lv *et al.* 2012). Currently, all of the reported LNV strains were isolated from China.

LNV can replicate in various mammalian cells and cause viremia and hemorrhage in mice, suggesting LNV maybe pathogenic to humans or animals (Attoui *et al.* 2006). However, there has been no confirmed report of human disease as a result of LNV in China.

#### *Yunnan orbivirus* (YNOV)

YUOV is a newly identified member of the genus *Orbivirus* within the family *Reoviridae*. The viral genome is composed of 10 segments with conserved terminal sequences (Attoui *et al.* 2005a; Liu *et al.* 2011). It was first isolated in 1999 from *Culex tritaeniorhynchus* in Lanchang County in Yunnan, China. Its presence has so far been reported in China, Australia, and Peru (Attoui *et al.* 2005a, 2009; Zhang and Liang 2012).

In recent years, six strains of YOUV have been isolated from *Culex tritaeniorhynchus* in the China-Myanmar-Laos border area (Wang *et al.* 2011). Sero-surveys have demonstrated YUOV IgM and IgG antibodies in febrile patients from the China-Myanmar-Laos border area, giving evidence of YUOV infection in the area.

#### *Tibet orbivirus* (TIBOV)

TIBOV represents the second orbivirus isolated from mosquito specimens in China. TIBOV (strain XZ0906) was first isolated from a pool of *Anopheles maculatus* mosquitoes in Tibet, China in 2009 (Li M *et al.* 2014). Subsequently, TIBOVs were isolated from *Culicoides spp*., *Culex fatigan*, or *Culex tritaeniorhynchus* mosquitoes collected from Yunnan or Guangdong (Lei *et al.* 2015; Xing *et al.* 2017). TIBOV could replicate in C6/36 and BHK-21 cells and cause CPE. Currently, it is unclear whether TIBOV can infect either humans or animals. Further studies should be worked for serological survey to define potential human and animal exposures to TIBOV.

### *Togaviridae*

#### *Chikungunya virus* (CHIKV)

CHIKV is a member of the genus *Alphavirus* and the family *Togaviridae* (Pialoux *et al.* 2007). The virus was first isolated in Tanzania in 1952, and since then CHIKV has spread to Asia, Africa, the Americas, and the areas surrounding the Indian and Pacific Oceans. In China, CHIKV was first isolated from the serum of a patient in Yunnan in 1987 (Huang *et al.* 1998). Based on the CHIKV E-gene, the virus has been phylogenetically classified into three distinct groups: the Asian genotype, the West African genotype, and the East/Central/Southern African (ECSA) genotype (Pialoux *et al.* 2007).

The main vectors for CHIKV are *Aedex aegypti* and *Aedex albopictus* mosquitoes, the same mosquitoes that transmit DENV (Pialoux *et al.* 2007). Other *Aedes* species are sensitive to experimental CHIKV infection, but their role in field transmission has not yet been shown.

Human beings are the known natural hosts of CHIKV. Serological evidence suggests that non-human primates, some rodents and birds, and other vertebrate species may serve as reservoir hosts in nature (McIntosh *et al.* 1964; Pialoux *et al.* 2007; Filipe and Pinto 1969; Sam *et al.* 2015). Recently, the experimental infection of 12 mammalian species and nine avian species indicated that some bats and rodents may serve as reservoir hosts of CHIKV; however, various domestic and wild animal species, including birds, are unlikely to be reservoirs of CHIKV in nature (Bowen *et al.* 2016).

The most common symptoms of CHIKV infection in humans are fever and joint pain; other symptoms may include headache, rash, nausea, and vomiting. Currently, there is no licensed vaccine or antiviral drug available for CHIKV infection. Although several sporadic cases of non-indigenous CHIKV infection have been documented in China, no outbreak was reported prior to the year 2010. For instance, the first imported sporadic CHIKV infection was reported in Xishuangbanna, Yunnan, in 1987 (Huang *et al.* 1998). Four imported cases of CHIKV infection were detected in Guangdong Province, in travelers returning from Sri Lanka and Malaysia in 2008 (Zheng *et al.* 2010). One imported case of CHIKV infection was reported in Zhejiang in 2012 (Sun *et al.* 2013). The first outbreak of chikungunya fever, with 129 laboratory-confirmed cases, was reported in Guangdong in 2010 (Zhang *et al.* 2012; Wu *et al.* 2012). The virus in this outbreak belonged to the CES genotype. This outbreak was considered a local outbreak of chikungunya fever caused by an imported case, although the source is unclear (Butt *et al.* 2014; Li *et al.* 2012; Lu X *et al.* 2014; Zhang *et al.* 2012).

#### *Getah virus* (GETV)

GETV is a member of the genus *Alphavirus*, family *Togaviridae*. GETV can cause disease in horses and pigs. It has not been linked to causing disease to humans. It was first isolated from *Culex gelidus* mosquitoes from Malaysia in 1955. Subsequently, GETV has been isolated in various countries such as Japan, the Philippines, the Republic of Korea, India, and China (Brown and Timoney 1998; Nemoto *et al.* 2016; Seo *et al.* 2012; Zhai *et al.* 2008a). Recent isolates of GETV in Russia, Mongolia, and China indicate a change in the world distribution in terms of re-emerging GETV (Zhai *et al.* 2008a).

In China, GETV was first (strain M1) isolated from *Culex* mosquitoes in Hainan province in 1964. Since then, various GETV isolates have been found in different mosquito species including *Aedex vexans*, *Armigeres obturbans*, *Armigeres subalbatus*, *Culex* spp. and unidentified mosquitoes in Hebei, Yunnan, Shanghai, Gansu, and Shanxi during the years 2002–2012, which indicates that GETV is distributed widely in China (Li *et al.* 2017; Liu *et al.*. 2011; Wang *et al.*. 2011; Zhai *et al.* 2008a; Zheng *et al.* 2015). The full-length sequences of the Chinese GETV isolates, South Korean GETV isolate, and *Sagiyama virus* (SAGV) (Japanese isolate) showed that these viruses exhibit high identity, and up to 5.5% amino acid sequence divergence was calculated between the original Malaysian isolate of GETV and the Chinese isolates (Zhai *et al.* 2008a). Neutralizing antibodies to GETV have been identified in serum samples from humans, horses, and pigs in Hainan province in China (Li *et al.* 1992).

#### *Ross River virus* (RRV)

RRV is a member in the genus *Alphavirus*, family *Togaviridae*, which can cause polyarthritis and arthralgias. RRV was first isolated from *Aedes vigilax* captured in the Australian Ross River region in 1959 (Doherty *et al.* 1968). RRV is widely distributed in the Pacific region (such as Fiji, Papua New Guinea, Cook Islands) and Australia (Harley *et al.* 2001; Klapsing *et al.* 2005; Rosen *et al.* 1981; Russell 2002; Scrimgeour *et al.* 1987; Yu *et al.* 2014).

In China, currently there is only one RRV isolate (strain HBb17) reported, which was isolated from brain tissue of bat in Hainan province. Experimental infection showed that HBb17 could replicate in mosquitoes and cause mice to die (Zhao *et al.* 1997). RRV-specific IgG antibodies were detected as positive in serum samples of healthy individuals and rats in Hainan province by immunofluorescence assay (IFA) in 1993 (Zhao *et al.* 1997). In addition, a sero-survey for viral encephalitis patients and healthy individuals during 2005–2008 in Guizhou indicated there was RRV infection in this region (Ye *et al.* 2012). However, no survey for the RRV prevalence in mosquitoes collected in China has been reported.

#### *Sindbis virus* (SINV)

SINV is a member of the western equine encephalomyelitis complex and the prototype species of the genus *Alphavirus* in the family *Togaviridae* (Laine *et al.* 2004). SINV (strain Eg339) was first isolated from *Culex univittatus* mosquitoes collected in Sindbis village in Egypt in 1952 (Taylor *et al.* 1955). SINV is widely distributed in the world, and strains are divided into three genotypes: Paleoarctic/Ethiopian (P/E), Oriental/Australian (O/A), and Western/Australian (W/A) (Taylor *et al.* 1955).

The first SINV (strain YN87448) in China was isolated in 1986 from the blood of a patient with fever in Yunnan Province (Zhou *et al.* 1999). To date, only four SINV isolates have been reported in China, three from Yunnan—YN87448, Sindbis-IMB (1992), and MX10 (2005)—and one from Xinjiang (Liang *et al.* 2000; Wang *et al.* 2008, 2011; Zhou *et al.* 1999). Phylogenetic analysis suggests that YN87448, Sindbis-IMB, and XJ-160 belong to the P/E genotype and XJ-160 is clustered in a separate clade. MX10 belongs to the O/A genotype (Liu *et al.* 2011; Zhang and Liang 2012).

SINV is maintained in nature through an avian-mosquito transmission cycle; the main vectors are *Culex spp.* and *Culiseta spp.* mosquitoes. Occasionally, humans and other vertebrates are infected (Kurkela *et al.* 2008; Laine *et al.* 2004). SINV infection causes fever, rash, arthritis, and on rare occasions encephalitis in humans, and the clinical infections are reported mainly in northern Europe and South Africa (Adouchief *et al.* 2016; Kurkela *et al.* 2008). In China, a sero-survey study demonstrated SINV-specific antibodies (IgG) detected in healthy individuals or patients with unknown fever, encephalitis, and animals (dogs, foxes, rabbits) in Yunnan, Hainan, Xijiang, Guangdong, and Fujian. This indicates that SINV might be causing fever and viral encephalitis in China (Liu *et al.* 2011; Zhang and Liang 2012).

## Mosquito-Specific Viruses

### *Flaviviridae*

#### *Chaoyang virus* (CHAOV)

CHAOV is a newly recognized ISV of the genus *Flavivirus* in the family *Flaviviridae*. Currently, there is no evidence to suggest a role for CHAOV in animal or human disease. CHAOV was first isolated (strain Deming) from *A. vexans* in Chaoyang city in Liaoning, China, in 2008 (Liu *et al.* 2011; Wang ZS *et al.* 2009). Currently, the isolation of CHAOV has only been reported in China and the Republic of Korea. An unknown flavivirus (ROK144), which was isolated from *Aedex vexans nipponii* collected in 2003 in the Republic of Korea, was subsequently determined to be CHAOV (Lee *et al.* 2013). Phylogenetic analysis with other flaviviruses showed that CHAOV should be grouped in the same clade as the insect-specific flaviviruses, Lammi virus (LAMV) and Donggang virus (DGV) (Takhampunya *et al.* 2014). In China, other strains of CHAOV (BeiBei and HLD115) were isolated in 2008 and 2010, respectively. CHAOV Deming strain was shown to induce cytopathic effect (CPE) in C6/36 cells, but cannot replicate in vertebrate cell lines such as baby hamster kidney (BHK), primary duck, primary chicken, and Vero cells (Lee *et al.* 2013; Liu *et al.* 2011; Takhampunya *et al.* 2014).

#### *Culex flavivirus* (CxFV)

CxFV is an ISV of the genus *Flavivirus*, family *Flaviviridae*. It was first isolated in 2003 in Japan from *Culex pipiens* and *Culex tritaeniorhynchus* (Hoshino *et al.* 2007). Subsequently, the virus has been isolated in Asia, Africa, and America (Cook *et al.* 2009; Wang *et al.* 2012; Kim *et al.* 2009; Machado *et al.* 2012). Phylogenetic analysis based on the *E* gene of CxFVs indicates that they can be divided into two distinct genotypes. CxFV (strain SDDM06-11) was first isolated from *Culex pipiens* mosquitoes collected in Shandong, China, in 2006 (Huanyu *et al.* 2012). A larger survey for the distribution and phylogenetic analysis of CxFV in China was conducted during 2004–2012 (Liang *et al.* 2015). The result showed 29 (29/871 pools) RNAs from *Culex pipiens*, *Culex tritaeniorhynchus*, *Anopheles sinensis*, and *Culex spp.* tested positive for CxFV. In addition, six CxFV strains were also isolated from *Culex* species from Shandong, Henan, and Shaanxi. Phylogenetic analysis of the E gene indicated that the Chinese strains formed a robust subgroup of genotype 1, together with viruses from the United States and Japan (Liang *et al.* 2015). In 2011, new CxFVs were isolated in Liaoning. In addition, Zuo *et al.* reported the isolation of CxFV in Yunnan during 2007–2010. In this study, 11 isolates for YNCxFV were obtained, 10 were from *Culex tritaeniorhynchus* and one was from *Anopheles sinensis*. The strains of YNCxFV were most similar to Quang Binh virus (QBV) VN180 isolated from Vietnam (Zuo *et al.* 2014).

### *Mesoniviridae*

Mesoniviruses are widely distributed in different geographic regions and have a large host range of mosquito species (Lauber *et al.* 2012; Thuy *et al.* 2013).

#### *Nam Dinh virus* (NDiV)

NDiV is a member of the genus *Alphamesonivirus* within the newly established family *Mesoniviridae*. NDiV has been successfully isolated from various mosquito species including *Culex vishnui*, *Culex tritaeniorhynchus*, *Culex pipiens quinquefasciatus*, and *Aedes albopictus* in Vietnam and the United States. NDiV was first isolated (strain SZ11706Z) in China from samples of *Culex pipiens quinquefasciatus* in Shenzhen, Guangdong, in 2011 (Zhou *et al.* 2017). Phylogenetic analysis indicated that the NDiV-CHN strain (SZ11706Z) is closely related to the NDiV-VIE strain (02VN178) isolated from *Culex tritaeniorhynchus* in Vietnam. NDiV-CHN was grouped into the type species *Alphamesonivirus 1*, which is represented by NDiV and Cavally virus (CavV) (Zhou *et al.* 2017).

#### *Yichang virus* (YCV)

YCV is a novel mosquito virus within the family *Mesoniviridae*. It was originally isolated from *Culex spp.* mosquitoes in Hubei, China, in 2014, and the prevalence of the YCV sequence in mosquitoes collected from Hubei was 16.5%. YCV has the largest genome size in the family *Mesoniviridae*, and phylogenetic analyses indicate it belongs to an unassigned genus (Wang *et al.* 2017).

### *Parvoviridae*

#### *Culex pipiens pallens densovirus* (CppDNV)

CppDNV belongs to the genus *Brevidensovirus* within the family *Parvoviridae*. CppDNV was first isolated from wild-caught adult female *Culex pipiens pallens* mosquitoes in Liaoning, China, in 2000 (Zhai *et al.* 2008b). The virus has subsequently been isolated from several species of mosquitoes, including *Culex pipiens quinquefasciatus*, *Culex tritaeniorhynchus*, and *Anopheles sinensis*, and other mosquitoes collected in various provinces of China such as Yunnan, Qinghai, Shanxi, and Inner Mongolia (Cao YX *et al.* 2011; Feng *et al.* 2014; Li *et al.* 2010; Zhai *et al.* 2008b; Zheng *et al.* 2015). Currently, no isolates of CppDNV have been reported outside China. Analysis of the phylogenetic relationships and the genome organization of CppDNV clearly showed that this virus clustered with the species *Aedes aegypti* densovirus (AaeDNV) and represented a novel variant of this species (Zhai *et al.* 2008b).

### *Reoviridae*

#### *Kadipiro virus* (KDV)

KDV, which was once classified as coltivirus JKT-7075, belongs to the genus *Seadornavirus* within the family *Reoviridae* (Attoui *et al.* 2000). KDV was first reported in Indonesia, and subsequently found in China. Five strains of KDV, which can cause CPE in C6/36 cells, were isolated from *Culex tritaeniorhynchus*, *Anopheles sinensis*, and *Armigeres obturbans* in northwestern Yunnan province in China in 2005 (Liu *et al.* 2011; Sun *et al.* 2009a, b).

#### *Mangshi virus* (MSV)

MSV is a newly discovered virus in the genus *Seadornavirus* of the family *Reoviridae* and phylogenetically close to BAV. The virus can cause CPE in C6/36 cells but not in mammalian BHK-21 or Vero cells. It was first isolated (strain DH13M041) from one pool of mosquitoes (*Culex tritaeniorhynchus*) collected in Mangshi City, Dehong Prefecture, southwest of Yunnan in China in 2013 (Wang J *et al.* 2015). Currently, there are no reports of MSV outside China.

## Summary

**Fig. 1:**
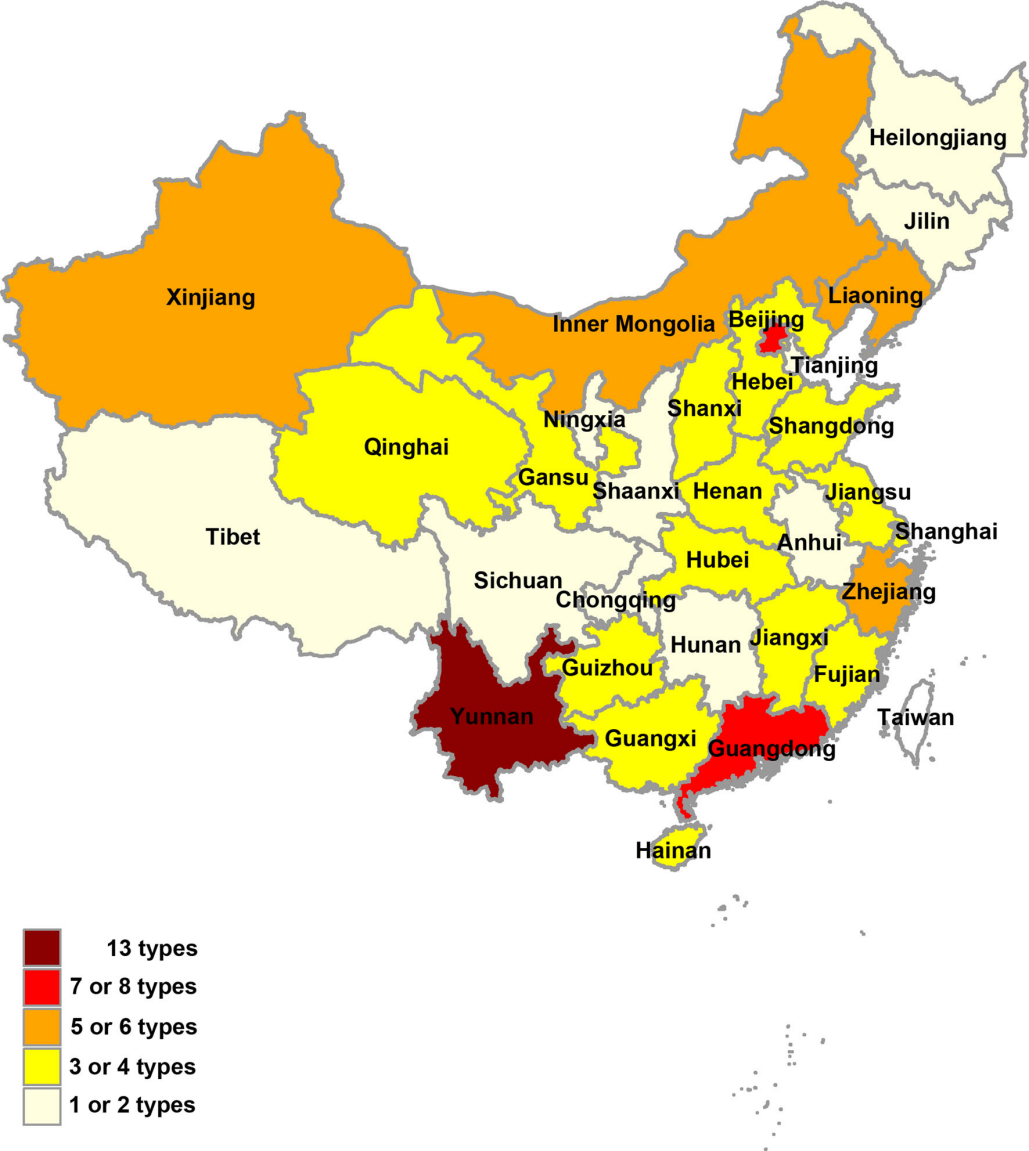
Distribution of mosquito-associated viruses across mainland China. There are 24 types of mosquito-associated viruses (Table 1) obtained from mosquitoes in different regions of China. The mosquito-associated viruses are highly prevalent in the regions of Yunnan, Beijing, Guangdong.

The relative distribution of mosquito-associated viruses in China is shown in Fig. 1. A high prevalence of mosquito-associated viruses exists in the regions of Yunnan, Beijing, Xinjiang, Inner Mongolia, Liaoning, Zhejiang, and Guangdong. The major viruses in high-incidence areas include DENV, JEV, and TMUV (Table 1). In addition, many mosquito-associated virus sequences such as Wuhan mosquito virus, Xinzhou mosquito virus, Zhejiang mosquito virus, Zhee mosquito virus, Wutai mosquito virus, *Culex tritaeniorhynchus* rhabdovirus, etc., belonging to various viral genera were found by deep sequencing of Chinese mosquito samples from Hubei and Zhejiang in recent studies, which suggests that more extensive cell culture efforts are highly likely to yield additional viruses (Li *et al.* 2015; Shi *et al.* 2015, 2016). With the current global warming challenge that is greatly influencing world climatic conditions, unintentional transfer of infected vectors, frequent human migration, rapid urbanization, and widespread deforestation, coupled with new technologies such as deep sequencing, the number of mosquito viruses discovered might increase greatly in China. In the future, we recommend that: (1) surveillance of mosquito-associated viruses, vectors, and hosts, especially viruses that can cause human diseases, should be continuous and be highly intensified; (2) as the association of some of the mosquito viruses such as BAV, YNOV, SINV, and TAHV to human diseases still remain unclear, further pathogenicity investigation studies should be conducted; and (3) in order to reveal why some mosquito-borne viruses possess the ability to infect and cause disease in humans, comparative studies between mosquito-specific viruses and medically important mosquito-borne viruses should be conducted.
